# Papillary Renal Carcinoma Arising in an Ectopic Native Kidney and Status after Renal Transplant: A Report of a Unique Case and Review of the Literature

**DOI:** 10.1155/2012/831403

**Published:** 2012-12-18

**Authors:** Xiangdong Xu, Noel Weidner

**Affiliations:** ^1^Department of Pathology and Immunology, Washington University in St. Louis, 660 South Euclid Avenue, P.O. Box 8118, St. Louis, MO 63110, USA; ^2^Department of Pathology, University of California San Diego, San Diego, CA 92103-8720, USA

## Abstract

Renal ectopia is an uncommon developmental defect of upper urinary tract. Except for hydronephrosis and urinary calculus formation, it is believed that ectopic kidneys are not more susceptible to diseases compared to the normally positioned kidneys. Primary renal carcinoma in ectopic kidneys is rarely observed. Our literature review identified eight cases in nontransplanted patients; seven were clear-cell carcinoma and one was papillary renal carcinoma. On the other hand, native kidneys of renal transplant patients are fifteen times more likely to develop renal carcinoma than those of nontransplanted patients. Renal malignancy has never been reported in native ectopic kidneys of transplant recipients. We report the first case of a papillary renal carcinoma in a native ectopic kidney of a 30 year-old female, six-year status after renal transplantation.

## 1. Introduction

Renal ectopia is a rare developmental defect, in which a mature kidney fails to reach its normal position in the renal fossa at the level of the upper lumbar spine. The causes include arrest during migration, migration beyond normal limit, metanephric ectopia, contralateral metanephros induced by wandering ureteric bud, and duplex Wolffian ducts [1]. The average incidence is approximately 1 in 900 and involves both genders similarly [2]. Renal ectopia is divided into simple or crossed forms [3]. Simple ectopia refers to aberrant location of kidneys on the ipsilateral sides, which is usually further subclassified by the locations, including pelvic, iliac, abdominal and thoracic ectopia. Thoracic kidneys are the least common subtype and only account for approximately 5% of all renal ectopic cases [4]. On the other hand, crossed ectopia is characterized with the ureter of the ectopic kidney crossing the midline before entering the bladder on the expected side. A majority of crossed ectopia is associated with fusion [3].

It is believed that ectopic kidneys carry similar risks of developing diseases compared to those of normally positioned kidneys, except for urinary calculus formation and hydronephrosis [2]. In terms of primary renal malignancies, there are only rare cases arisen in ectopic kidneys. Eight cases were found in the post-CT era: seven were clear cell carcinoma and one was papillary renal carcinoma (PRCC) [5–11]. So far, there is no reported primary renal malignancy arising in native ectopic kidneys in renal transplant patients, even though the transplant history is associated with a fifteen-time higher risk of renal cancers on the native kidneys [12].

Here we report the first case of renal cell carcinoma arisen in a Bochdalek ectopic kidney of a post renal transplant patient, secondary to end stage renal disease. 

## 2. Case Presentation

The patient is a 30-year-old female with a history of nonfunctioning right ectopic kidney associated with congenital Bochdalek-type diaphragmatic hernia, status after surgical repair after birth. Imaging studies show that her right ectopic kidney locates within a Bochdalek-type diaphragmatic hernia. The ectopic kidney showed abnormal rotation with the long axis in the transverse plane and the renal hilum facing posterior. Along with the ectopic kidney, adrenal gland and retroperitoneal fat are also displaced cephalad into the lower chest. Additional developmental abnormalities include rotational abnormality of the intestines, uterus, and ovaries.

She was diagnosed with severe hypertension and acute renal failure during early adulthood. Her renal function continued to deteriorate and consequently progressed to chronic renal failure. She underwent peritoneal dialysis, hemodialysis, and eventually had renal transplantation. Six years after renal transplantation, an incidental 2.3 cm renal mass was found in the right ectopic native kidney on MRI abdomen, suspicious for renal cell carcinoma. The patient underwent right radical nephrectomy. 

The specimen received in the gross room was an atrophic kidney. Sectioning revealed a single-well-circumscribed, tan, light-yellow renal mass 2.6 × 2.5 × 2.0 cm, in the upper pole. The tumor abutted renal capsule. Grossly, there were tan, yellow-colored tiny dots and streaks noted within the tumor. No hemorrhage, necrosis, or cystic degeneration was identified grossly. No urinary calculus was identified in renal pelvis.

**Figure 1:**
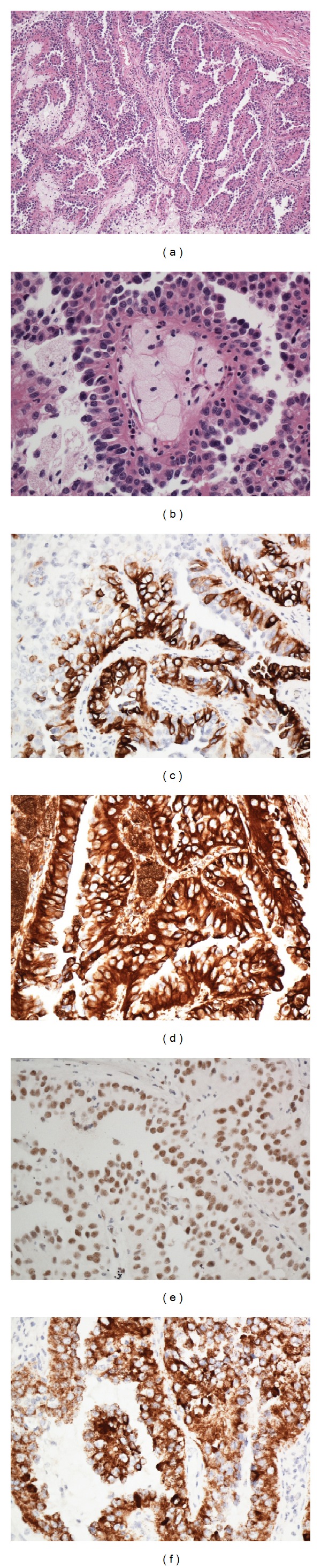
Histological and immunohistochemical features of the papillary renal cell carcinoma. Low-power view shows the papillary structures with fibrovascular cores ((a), original magnification 100x). The columnar neoplastic cells have finely granular eosinophilic cytoplasm, bland nuclear feature. Foamy histiocytes are seen within the fibrovascular cores ((b), original magnification 400x). The neoplastic cells are positive for CK7 (c), vimentin (d), PAX-2 (e), and AMACR (f) (original magnification 400x).

Microscopically, the tumor was well circumscribed and surrounded by dense fibrous band. The surrounding renal parenchyma showed typical features of “end-stage kidney,” such as thick-walled arterioles and “thyroidized” tubules. No epithelial atypia or premalignant changes were seen in the surrounding parenchyma. The neoplasm had a prominent papillary pattern with true fibrovascular cores. The papillae were lined with pseudostratified columnar neoplastic cells with uniform round-to-oval nuclei and finely granular eosinophilic cytoplasm. Frequent foamy histiocytes were identified within the fibrovascular cores (Figures 1(a) and 1(b)). The neoplastic cells were positive for CK7 (Figure 1(c)), vimentin (Figure 1(d)), PAX-2 (Figure 1(e)), and AMACR (Figure 1(f)). Additional immunoreactive markers include CK19, CD10 (focally and weakly), E-cadherin (focally), and EMA (data not shown). No immunoreactivity was observed for CK20 or RCC (data not shown). The overall findings were consistent with papillary renal cell carcinoma, type 2.

## 3. Discussion

PRCC comprises approximately 10% of renal cell carcinoma. It commonly occurs in 5th to 6th decades with a slight male predilection. Multifocal and/or bilateral tumors are more commonly seen in PRCC than in other renal parenchymal malignancies. Histologically, PRCC is characterized by papillary or tubulopapillary architecture lined by neoplastic cells. There are two morphologic variants of PRCC. In Type 1 PRCC, the neoplastic cells are usually small with scanty cytoplasm and arranged in a single layer over the papillae. In Type 2 PRCC, the neoplastic cells often show pseudostratification and have more eosinophilic cytoplasm than type I PRCC [13]. The morphology of our case is consistent with type 2 PRCC. 

The simple renal ectopia is further divided into pelvic, iliac, abdominal, and thoracic kidneys based on the location, among which thoracic kidney is the least common subtype. Different methods have been proposed to categorize thoracic ectopic kidneys. For example, Stephens et al. suggested four subtypes of thoracic, or superior kidneys: supradiaphragmatic, transdiaphragmatic, infradiaphragmatic, and Bochdalek ectopic kidney [1]. Based on the clinical history and imaging findings, our patient has a thoracic kidney associated with Bochdalek-type congenital diaphragmatic hernia (CDH). 

CDH is defined as an abnormality in the integrity of the diaphragm, including discontinuity (hole) and undermuscularization (eventration). The prevalence of CDH is 1 per 4000 births [14]. Based on anatomic locations, CDH is classified into Bochdalek (90% of all cases) and non-Bochdalek hernias. Alternatively, CDH can also be divided into isolated and complex CDH. Complex CDH has additional major abnormalities, such as well-defined developmental syndromes, identifiable chromosome abnormalities, or a nonsyndromic constellation of major malformations [14]. Our patient has no other major malformations observed. There is no information available about any chromosomal abnormalities or any CDH cases in relatives of this patient. Therefore, our case is most likely an isolated Bochdalek-type CDH with associated “nonmajor” abnormalities.

Renal parenchymal malignancies in ectopic kidneys are extremely rare and there are only 8 cases reported in the literature since 1995 (Table 1). All of the eight patients are middle-aged males with a median age of 52-year old. None of the patients had kidney transplant. The types of renal ectopia include pelvic (2 cases), crossed fused (5 cases), and crossed solitary kidney (1 case). Types of renal malignancies include clear renal cell carcinoma (4 cases), renal cell carcinoma with no further specification (3 cases), and papillary renal cell carcinoma (1 cases).

It is known that renal-transplanted patients have higher risks of many different malignancies, probably due to the long duration of immunosuppression. Specifically, native kidneys of renal transplant recipients have 15 times and 1.4 times significantly higher risk of developing renal cancers, compared to those of normal population and to those of patients on transplant waiting list, respectively [12]. The majority of renal cancers arisen in native kidneys of renal transplant recipients are incidental findings and low-grade tumors, more often PRCC, and usually have a favorable prognosis [15]. Arising in an ectopic kidney, the PRCC of our case is an incidental finding, and small in size, recapitulating typical clinicopathologic features of RCC derived from native kidneys of transplanted patients.

In summary, we report a unique case of renal parenchymal malignancy arising in a Bochdalek-type thoracic kidney, six-year status after renal transplantation.

## Figures and Tables

**Table 1:** Eight cases of renal parenchymal malignancies in ectopic kidneys.

ID	Age/gender	Type of malignancy	Type of renal ectopia	Reference
1	46/M	CC-RCC	Solitary, crossed pelvic ectopic	Grotas and Phillips [11]
2	50/M	RCC	Crossed fused	Basoglu et al. [6]
3	51/M	CC-RCC	Crossed fused	Romero et al. [9]
4	52/M	RCC	Pelvic	Terrone et al. [8]
5	52/M	CC-RCC	Pelvic	Coşkun et al. [5]
6	53/M	PRCC	Crossed fused	Davis et al. #1 [10]
7	60/M	CC-RCC	Crossed fused	Davis et al. #2 [10]
8	62/M	RCC	Crossed fused	Stimac et al. #5 [7]

RCC: renal cell carcinoma; CC-RCC: clear cell renal cell carcinoma; PRCC: papillary renal cell carcinoma.
